# Conducting public health surveillance in areas of armed conflict and restricted population access: a qualitative case study of polio surveillance in conflict-affected areas of Borno State, Nigeria

**DOI:** 10.1186/s13031-022-00452-2

**Published:** 2022-05-07

**Authors:** Eric Wiesen, Raymond Dankoli, Melton Musa, Jeff Higgins, Joseph Forbi, Jibrin Idris, Ndadilnasiya Waziri, Oladapo Ogunbodede, Kabiru Mohammed, Omotayo Bolu, Gatei WaNganda, Usman Adamu, Eve Pinsker

**Affiliations:** 1 US Centers for Disease Control and Prevention, Atlanta, USA; 2 World Health Organization, Maiduguri, Borno State, Nigeria; 3 National Stop Transmission of Polio, Abuja, Nigeria; 4 National Primary Health Care Development Agency, Abuja, Nigeria; 5 University of Illinois at Chicago, Chicago, USA

## Abstract

This study examined the impact of armed conflict on public health surveillance systems, the limitations of traditional surveillance in this context, and innovative strategies to overcome these limitations. A qualitative case study was conducted to examine the factors affecting the functioning of poliovirus surveillance in conflict-affected areas of Borno state, Nigeria using semi-structured interviews of a purposeful sample of participants. The main inhibitors of surveillance were inaccessibility, the destroyed health infrastructure, and the destroyed communication network. These three challenges created a situation in which the traditional polio surveillance system could not function. Three strategies to overcome these challenges were viewed by respondents as the most impactful. First, local community informants were recruited to conduct surveillance for acute flaccid paralysis in children in the inaccessible areas. Second, the informants engaged in local-level negotiation with the insurgency groups to bring children with paralysis to accessible areas for investigation and sample collection. Third, GIS technology was used to track the places reached for surveillance and vaccination and to estimate the size and location of the inaccessible population. A modified monitoring system tracked tailored indicators including the number of places reached for surveillance and the number of acute flaccid paralysis cases detected and investigated, and utilized GIS technology to map the reach of the program. The surveillance strategies used in Borno were successful in increasing surveillance sensitivity in an area of protracted conflict and inaccessibility. This approach and some of the specific strategies may be useful in other areas of armed conflict.

## Introduction

### Global strategy

The Global Polio Eradication Program was established in 1988 with the lofty goal of eradicating polio globally by the year 2000 [1]. The key strategies for polio eradication are achieving high coverage with 4 doses of polio vaccine for all infants, supplemental polio mass vaccination campaigns to boost polio immunity, sensitive surveillance to rapidly detect poliovirus circulation, and outbreak response vaccination campaigns. Since its inception, the program has reduced the annual incidence of paralytic polio from over 350,000 cases in 125 countries in 1988 to only 140 cases in just two countries in 2020 [2]. Despite this remarkable progress, the program is now 20 years past the target date and struggling to stop transmission in the remaining indigenous wild poliovirus reservoirs [3] in parts of Pakistan and areas of conflict in Afghanistan.

### Requirements for polio surveillance

Sensitive poliovirus surveillance is a key component of the effort to eradicate polio because it allows the program to rapidly detect and respond to any cases of polio to stop the transmission [4]. The poliovirus surveillance system is centered primarily on active surveillance for any case of acute flaccid paralysis (AFP) in children with laboratory testing of fecal specimens for poliovirus. Conducting this surveillance well requires a comprehensive network of district surveillance officers and health facility surveillance focal persons to quickly detect, report, and investigate AFP cases as they occur [4]. This network requires participation by public and private health care providers and is often augmented with support from partners such as the World Health Organization (WHO). There are a set of performance indicators that track the functioning and sensitivity of typical AFP surveillance systems. However, in areas of armed conflict these surveillance systems are challenged.

### Challenges in surveillance in armed conflicts

The ability to conduct sensitive surveillance is substantially curtailed in situations of insecurity and inaccessibility due to armed conflict [5]. Without full access to the population for vaccination and surveillance, poliovirus can circulate undetected. For example, an outbreak of polio in South Sudan was detected in 2008, which, based on poliovirus genomic sequencing analysis,^1^ [7], represented three years of undetected transmission due to ongoing conflict in that country [8]. Disruptions to both vaccination and surveillance have led to polio outbreaks and delayed detection in Afghanistan, Somalia, Angola, and the Democratic Republic of Congo as well [9].

### Context in Borno State, Nigeria

This complex problem, which can have far-reaching implications, is exemplified in the northern Nigeria State of Borno where wild poliovirus (WPV) was detected in 2016 and linked to transmission of lineages last detected in 2011, representing five years of undetected transmission due to the ongoing conflict in the state [10]. For over a decade Northeast Nigeria, and particularly Borno state, has been plagued by ongoing attacks by Boko Haram and offshoot terrorist groups [11]. These armed groups are responsible for mass killings, hostage takings, and destruction of houses and infrastructure including health facilities. During 2014–2016, Boko Haram gained control of progressively more territory in the state. Approximately 2.2 million people fled their homes due to the terrorist activities and millions more are in need of humanitarian assistance [12]. A large, number of people remained trapped in inaccessible areas of Borno State that the polio program could not access to conduct disease surveillance or vaccination [13]. Because of this situation, the polio program in Nigeria could not rule out the possibility of continued polio transmission in the state.

This study examined the impact of armed conflict on public health surveillance systems, the limitations of traditional surveillance strategies in this context, and potential strategies to overcome these limitations. The primary question was: how can the conventional polio surveillance system and strategies be modified for areas of conflict and inaccessible populations? Secondary questions focused on exploring the inhibitors of effective surveillance in the context of armed conflict, potential strategies to overcome them, modified performance monitoring mechanisms, and systems for facilitating collaboration for surveillance.

## Methods

### Study design

This study employed a qualitative single case study design to examine the AFP surveillance system in inaccessible areas of Borno State, Nigeria. Inaccessibility was defined as the inability of civilians to safely move in and out of a given area due to the risk of attack by insurgents. Elements of case study research include corroboration of findings from different types of evidence, use of a conceptual framework to guide the research design, and use of appropriate data collection and data analysis techniques to address issues of validity and reliability [14]. This design was chosen to allow an in-depth exploration of the challenges and strategies at play in a severe conflict situation.

## Researcher characteristics

The corresponding author conducted all the interviews and analyses for this study. Based in Atlanta, he had travelled to Borno state twice prior to the study to support the polio eradication program and had met some of the respondents. He was not working on polio eradication in Nigeria at the time of this study and did not attempt to bias or sway them in any way from providing their own perspectives during the interviews. While the use of a sole researcher to collect data is a limitation of the study, several strategies to strengthen validity were employed. These included: (1) triangulation among data sources—a document review was conducted prior to interviews, not to limit the interviews but to make it possible to explore, expand on, and question barriers and enablers tentatively identified through documents; (2) use of a second coder to check the researcher’s application of the codes, until 80% agreement was consistently reached; (3) member checking with the interview respondents to review the analysis and ensure that it accurately reflected their responses; (4) peer debriefing and discussion of results with a group of colleagues who work on polio eradication in areas of armed conflict in Nigeria, Somalia, Pakistan, and Afghanistan. Observations from member checking and peer debriefing [15] were incorporated into the final discussion of the data and greatly enriched the analysis.

### Conceptual framework

**Fig. 1:**
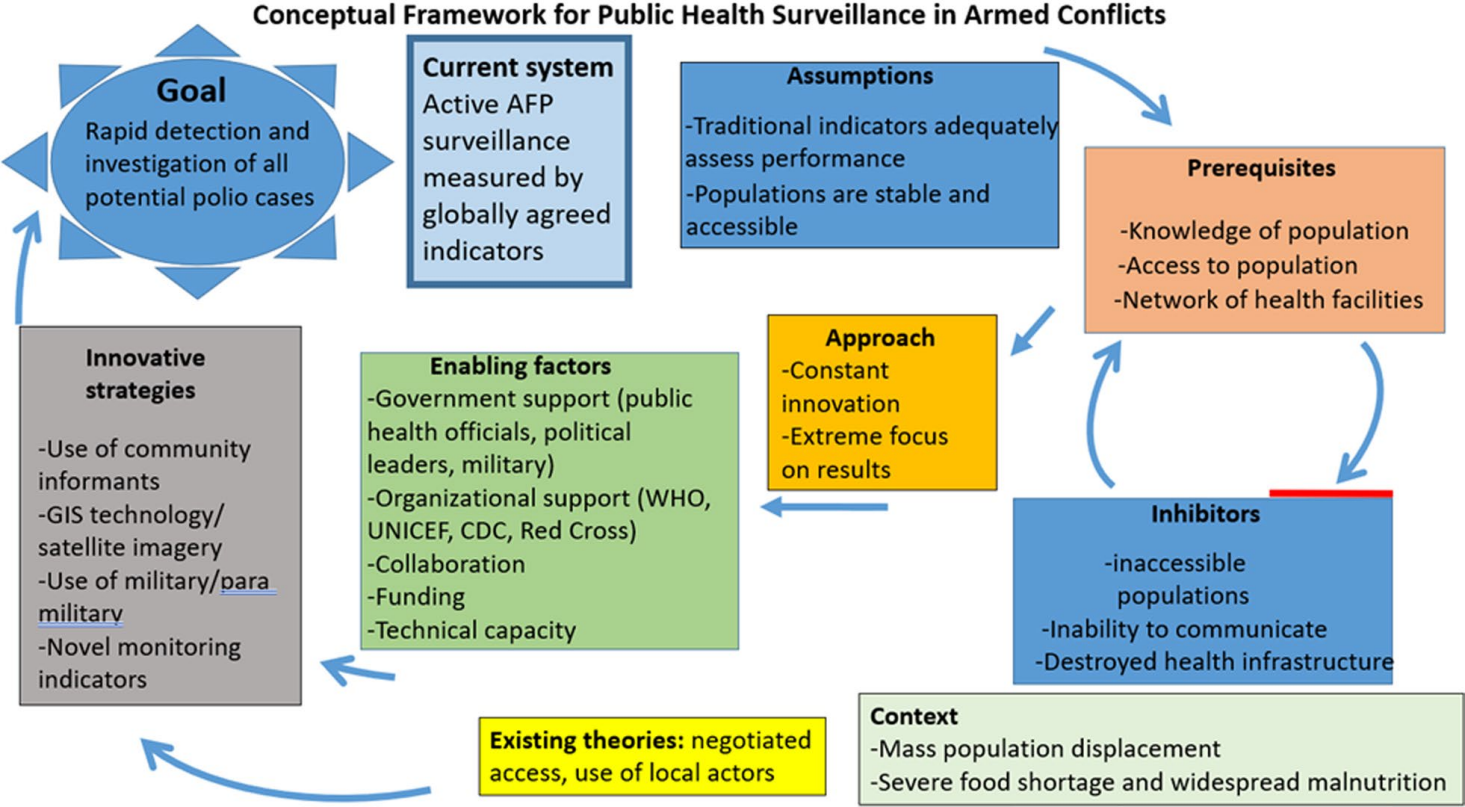
Conceptual Framework

A conceptual framework (Fig. 1) was developed (as Yin recommends for case studies as noted above) encompassing the key factors that affect the AFP surveillance system in conflict-affected areas, and revised following data analysis. The framework was developed though a review of the literature, analysis of the current polio surveillance structure, and reflections on the unique challenges affecting surveillance in Borno [16–29]. It includes the systems, assumptions, barriers, theories, and opportunities regarding conducting high quality polio surveillance in conflict-affected areas. The framework identifies the interconnections between these factors to focus on the opportunities to change the current system in ways that will make it more effective in the context of armed conflict. It illustrates the ways in which the current polio surveillance system is hindered in areas of armed conflict and suggests alternatives that may be effective in overcoming those barriers. Finally, it includes novel strategies such as engagement with local communities, engagement with security forces, use of tailored surveillance indicators, and use of remote sensing to assess population dynamics.

### Study sample

The sample for this study was purposefully selected [30] and encompassed 15 key documents and 16 staff selected for in-depth interviews. The sample strategy was designed to obtain a range of perspectives and experiences and to encapsulate both the field level (district and community) and the higher levels (state and national). The strategy also entailed reaching out to various organizations to understand the issues related to collaboration and information sharing. Finally, the strategy involved selecting various cadres of staff who are familiar with diverse aspects of surveillance including oversight functions, field surveillance, and data analysis. The list of potential staff to interview included 17 staff at the state and national level and 30 staff at the field level. We limited the interviews to a maximum of 30 people to ensure feasibility of the data collection. We purposively chose from among the staff in the sample to obtain a range of perspectives. In order to ensure a diversity of respondents, the sample included at least 3 staff from the district level and staff from at least 3 different agencies. We continued sampling until reaching a point of saturation in which we were hearing the same information and not gaining new insights from subsequent interviews. However, our study has limitations given that not everyone selected for the study opted to participate and there may have been some sample bias due to the participation levels. Key documents were selected to obtain detailed information on the armed conflict in Borno, the humanitarian response to the conflict, and polio eradication program activities in those areas. Documents included published reports, journal articles, program plans, and news media articles (Table 1).

**Table 1:** List of documents reviewed

Type of document	Document names	Date of publication	URL
Report	Displacement Tracking Matrix	December 2017	https://www.humanitarianresponse.info/en/operations/nigeria/assessment/displacement-tracking-matrix-dtm-round-xx-i-december-2017
Report	Nigeria Emergency Response. Borno State Early Warning Alert and Response System	October 2016	https://www.humanitarianresponse.info/sites/www.humanitarianresponse.info/files/documents/files/borno_state_weekly_epidemiological_bulletin_-_w41_2016_-_10_-_16_october.pdf
Report	Northeast Nigeria Humanitarian Response bulletin. Borno State Government	October 2016	https://www.humanitarianresponse.info/sites/www.humanitarianresponse.info/files/documents/files/borno_health_sector_bulletin_issue_number_1_sept_2016.pdf
Report	Northeast Nigeria Humanitarian Response bulletin. Borno State Government	July 2019	https://reliefweb.int/sites/reliefweb.int/files/resources/health_sector_bulletin_july_19_ne_nigeria.pdf
Report	Lake Chad Basin Crisis Overview	February 2016	https://reliefweb.int/report/nigeria/lake-chad-basin-crisis-overview-29-february-2016
Report	Global Polio Eradication Initiative (GPEI) polio updates for Borno 20016–2019	March 2020	http://polioeradication.org/
Report	33rd Nigeria Polio Expert Review Committee Report	January 2017	http://polioeradication.org/wp-content/uploads/2017/03/finalreport-33ERCmeeting-012017.pdf
Report	Finding inhabited settlements and tracking vaccination progress… in Borno	May 2019	https://pubmed.ncbi.nlm.nih.gov/31096971/
Report	Polio Independent Monitoring Board Report	October 2018	http://polioeradication.org/wp-content/uploads/2018/11/20181105-16th-IMB-Report-FINAL.pdf
Report	USAID Lake Chad Basin Complex Emergency Fact Sheet	May 2017	https://www.usaid.gov/sites/default/files/documents/1866/05.12.17_-_USAID-DCHA_Lake_Chad_Basin_Complex_Emergency_Fact_Sheet_15.pdf
Plan	National Primary Health Care Development Agency National Polio Eradication Emergency Plan	2018	http://polioeradication.org/wp-content/uploads/2018/04/Nigeria-National-Polio-Emergency-Plan-2018.pdf
News Article	New York Times article on Boko Haram: “Boko Haram is Back. With Better Drones.”	September 2019	https://www.nytimes.com/2019/09/13/world/africa/nigeria-boko-haram.html
News Article	Washington Post article on Borno conflict: “Nigerian children who escaped Boko Haram say they faced another ‘prison’: Military detention”	September 2019	https://www.washingtonpost.com/world/africa/nigerian-children-who-escaped-boko-haram-say-they-faced-another-prison-military-detention/2019/09/14/e30a0da2-d40c-11e9-8924-1db7dac797fb_story.html
Guidelines	WHO–recommended standards for surveillance of selected vaccine-preventable diseases*- polio	2018	https://www.who.int/immunization/monitoring_surveillance/burden/vpd/WHO_SurveillanceVaccinePreventable_18_Polio_R2.pdf?ua=1
Guidelines	Analyzing disrupted health sectors, a modular manual 2009. (Modules 2 and 4)	2009	https://www.who.int/hac/techguidance/tools/disrupted_sectors/en/

The staff interviewed in this study were selected to obtain a range of perspectives from various organizations, position types, and levels that are important for the functioning of the surveillance system (Table 2). The primary aim of these interviews was to gain a deeper understanding of the enablers and barriers for effective surveillance and the monitoring and collaboration systems. Five of the staff interviewed worked in four Local Government Areas (LGAs, districts) with high levels of conflict and inaccessibility: Guzamala, Bama, Ngala, and Kukawa. Interviews were continued until reaching a point of saturation where very little new information was gained from additional interviews.

**Table 2:** Interviews conducted

Number	Level	Organization	Position type
1	State	CDC contractor	Surveillance
2	State	CDC contractor	Surveillance
3	State	NSTOP	Surveillance
4	State	NSTOP	Surveillance
5	State	NSTOP	Surveillance
6	State	NSTOP	Data analysis
7	State	WHO	Surveillance
8	State	Ehealth	Data analysis
9	State	Solina	Data analysis
10	State	IOM	Humanitarian Support
11	District	NSTOP	Surveillance
12	District	NSTOP	Surveillance
13	District	NSTOP	Surveillance
14	District	NSTOP	Surveillance
15	District	MoH	Surveillance
16	International	CDC	GIS Specialist

CDC contractor, International contract with Centers for Disease Control and Prevention; WHO, World Health Organization; NSTOP, National Stop Transmission of Polio program (a cohort of trained health professionals recruited within Nigeria, modeled after the CDC-supported international STOP program [31]; Ehealth, Nigeria-based public health non-governmental organization; Solina, Nigeria-based health consulting firm; MoH, Ministry of Health

### Data collection and data management

Standardized interview guides were developed, pre-tested for clarity and relevance with relevant stakeholders and refined prior to data collection. Separate interview guides were developed for state level and district level interviews. Interviews were conducted between April and August 2020. All semi-structured telephone interviews were recorded and manually transcribed. Each interview was approximately one hour in length. Interview transcriptions were reviewed and cleaned for transcription errors prior to analysis. Reflective memos were produced immediately after each interview.

### Data analysis

Data relevant to the study questions and constructs in the 15 documents were extracted using a tool in Microsoft Excel© to create a matrix for analysis by construct and document type. The document extracts were analyzed to better understand the constructs in the study, identify emerging themes, and assess consistency of information among reports as a measure of the reliability of the available data.

Interview data were analyzed using MaxQDA© 2018 software [32]. Data were coded using both on *a-priori* and emergent codes (i.e. grounded in the data) (Table 3) following a hybrid approach to coding [33, 34]. Two rounds of coding were conducted to ensure that emerging codes and co-occurring codes were fully captured. Analysis was conducted using analytic memos, matrix displays, summary tables, code relations graphs, and code mapping to develop and describe themes and relationships in the data. Content from co-occurring codes was analyzed in further detail through summary tables and grids by organization. Results were compared and contrasted among respondents and respondent groups and also triangulated among interview data and reviewed documents to look for areas of convergence and divergence. Data analysis was conducted concurrently with data collection.

**Table 3:** Coding system

1 Inhibitors	2 Strategies	3 Monitoring systems	4 Collaboration and information sharing systems
1.1 Accessibility of populations	2.1 Community informants	3.1 Tailored surveillance performance indicators	(no subcodes were included for this code)
1.2 Communication	2.2 GIS technology	3.2 Tailored surveillance quality assessment tools	
1.3 Health infrastructure	2.3 Collection and testing of specimens beyond AFP cases		
1.4 Overall infrastructure	2.4 Collaboration with security		
1.5 Population movement	2.5 Profiling of displaced people		
1.6 Traumatizing violence	2.6 Evacuation (emerging code)		
1.7 Malnutrition and disease outbreaks	2.7 Nomadic population (emergent code)		
1.8 Rainy season (emergent code)			
1.9 Nomadic population (emergent code)			

### Ethical considerations

This study posed little risk to the participants. Only program staff who were already deeply involved in the issues were included. Our study design purposely excluded interviewees who could have been exposed to personal risk from participation in the study, e.g. identified community informants. Informed consent was obtained from each participant prior to conducting the interviews. All responses were kept confidential and no identifying information was retained electronically. Ethical approval was obtained from the Government of Borno State Ethical Review Board, and the case study was determined non-research by the CDC Center for Global Health Human Subjects Research Office and the University of Illinois Ethical Review Board. The researchers affirm that ethical considerations must be paramount in a study of this nature, both in conducting the study and in drawing recommendations from the results for potential application in other conflict-torn areas, e.g. considering what risks there may be to local populations in involving particular actors such as local informants or the military in surveillance.

### Role of the funding source

Funds provided by the US Centers for Disease Control and Prevention (CDC) were used to cover the cost of transcribing the interviews. CDC also allowed the primary investigator (a CDC employee) to work on this project during his working hours. The primary investigator conducted the study and made the decision to submit the manuscript for publication while working for CDC. CDC as an agency did not provide input into the study design or analysis.

## Results

**Fig. 2:**
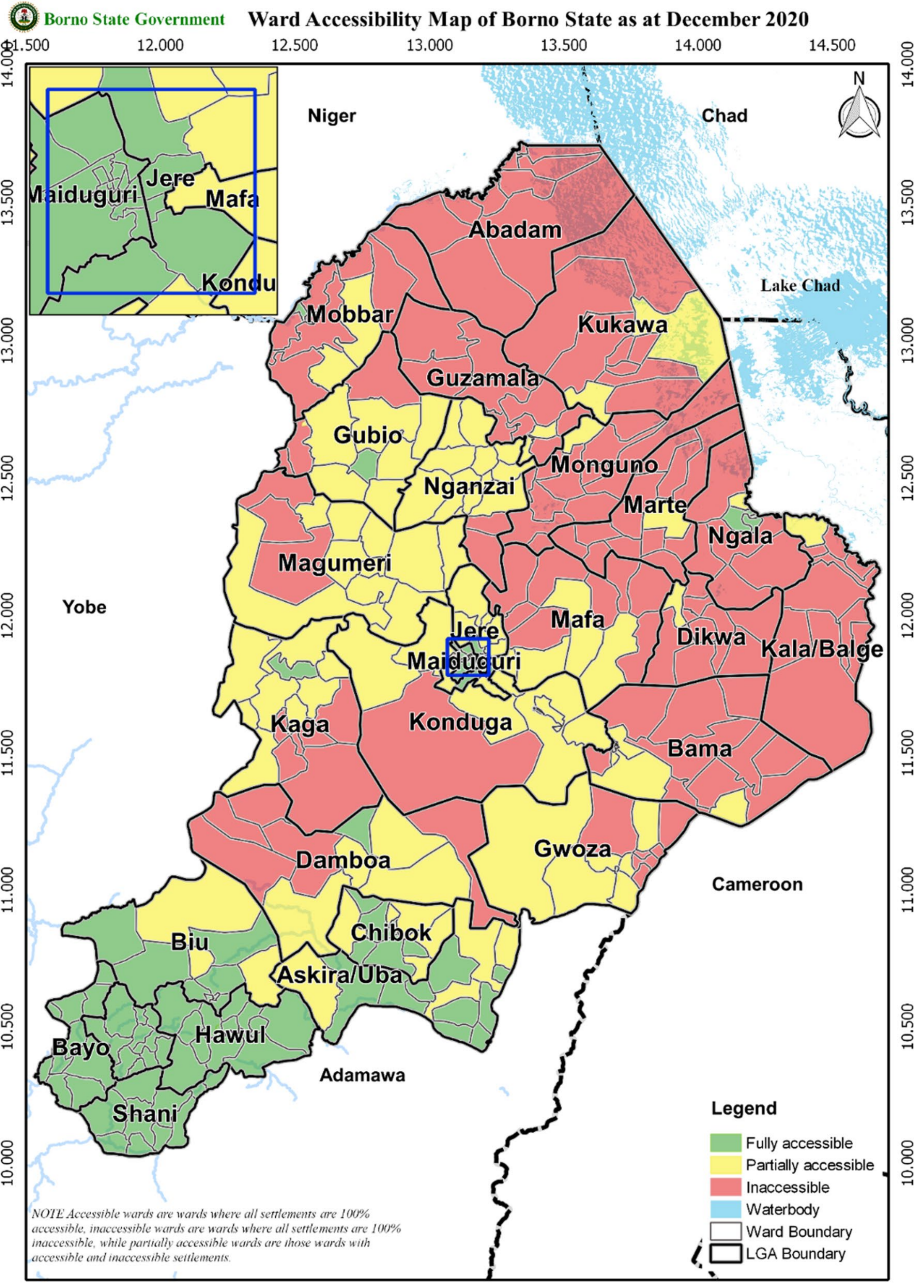
Accessibility in Borno State, December 2020 (provided by the Borno polio Emergency Operations Center)

Findings from the document review and interviews were consistent; the respondents’ interviews were very consistent and highly detailed (Table 4). The main inhibitors of surveillance in the conflict areas of Borno State were inaccessibility, and the destruction of both the health care infrastructure and the communication network; respondents unanimously reported that there were no functional health facilities and no cellular network in those areas. The traditional polio surveillance system relies on active surveillance in facilities, passive reporting, and prompt communication and could not function in the inaccessible areas. Figure 2 displays the accessibility by ward (sub-district) in Borno state as of December 2020. Other important challenges to the traditional AFP surveillance system, including traumatizing violence and widespread malnutrition, were considered surmountable. Population movement was viewed as a potential surveillance advantage because migrating families were primarily fleeing inaccessible areas to accessible areas, where they could more easily be captured in the surveillance system.

*Respondent 3: “Up to 45% of the state geographic area remain inaccessible. Take for example, there are 27 local governments in the state, only 6 are fully accessible” …”populations living in those areas cannot be reached by the regular teams that conduct AFP surveillance and surveillance for other vaccines preventable diseases. So, some populations are trapped there”*

*Respondent 1: “So, all those health facilities in those trapped communities have been destroyed.”*

*Respondent 4: “in those inaccessible areas, communication structures has been destroyed, so GSM networks*^2^*are not available. You won't be able to communicate on phone in those areas.”*

**Table 4:** Triangulation of data from document reviews and interviews

Construct	Sub Construct	Documents	Interviews	Level of Agreement
Inhibitors	Inaccessibility	Discussed by most*	Discussed by most	High
	Communication	Discussed by one	Discussed by most	High
	Health Infrastructure	Discussed by some	Discussed by most	High
	Overall Infrastructure	Discussed by one	Discussed by most	High
	Population movement	Discussed by most	Discussed by most	High
	Traumatizing violence	Discussed by some	Discussed by most	High
	Malnutrition and disease outbreaks	Discussed by most	Discussed by most	High
	Rainy season	Not discussed	Discussed by most	NA*
	Nomadic population	Not discussed	Discussed by some	NA
Strategies	Community informants	Not discussed	Discussed by most	NA
	GIS technology	Discussed by some	Discussed by most	High**
	Collection and testing of specimens beyond AFP cases	Discussed by some	Discussed by most	High
	Collaboration with security forces	Discussed by some	Discussed by most	High
	Profiling of displaced people	Discussed by some	Discussed by some	High
	Evacuation	Not discussed	Discussed by most	NA
	Nomadic population	Not discussed	Discussed by some	NA
Monitoring systems	Tailored surveillance performance indicators for inaccessible areas	Not discussed	Discussed by most	NA
	Tailored surveillance quality assessment tools	Not discussed	Discussed by some	NA
Collaboration and information sharing systems		Discussed by some	Discussed by most	High**

Note: “discussed by most” means that the majority of the documents or interviewers discussed the construct. “Discussed by some” means that at least two (but less than half) discussed it. High agreement means that the information was consistent with good agreement in the information provided. For example, for the inaccessibility construct, there was agreement across documents that there were many thousands of inaccessible people in many thousands of inaccessible settlements, and also that the size of the inaccessible population decreased considerably the over time from 2016–2018. While the exact numbers differ slightly, this aligns well with the information provided by the interview respondents

*Not applicable

**Interviewees provided additional information not found in the documents

Three strategies were found to be effective in overcoming these challenges: (1) use of local community informants to conduct surveillance in inaccessible areas; (2) local-level negotiation with insurgency groups to bring children with paralysis to accessible areas for investigation and sample collection; and (3) use of GIS technology to estimate the size and location of the population in inaccessible places (satellite imagery) and to track progress in surveillance. Together, these provided strong cumulative evidence of the absence of WPV transmission in Borno state. Other strategies discussed, but not emphasized by the respondents, were collection and testing of stool specimens from healthy children from inaccessible areas, collaboration with security forces, profiling newly arrived displaced persons, and accessing nomadic populations for surveillance.

### Use of local community informants for surveillance

Lay adults who resided in or were able to enter inaccessible areas were recruited as community informants in inaccessible areas (CIIAs) to search for children with suspected AFP. CIIAs were recruited through a snowball approach and included hunters, traders, nomads, and others identified at markets who were uninvolved in government programs, to protect them from anti-government sentiment. No stipend was provided; CIIAs were given an allowance after attending monthly meetings. The settlements they visited depended on whether they could indeed negotiate access. Their exact activities depended on the security risk level in the areas they reached, from simply observing children to directly asking adults if they had any paralyzed children in their or neighboring households. A separate coordination system to monitor CIIAs was set up with ward and LGA coordinators who were also intentionally distanced from the polio program to protect them from anti-government sentiment. Respondents agreed that CIIAs were reaching most, but not all settlements in inaccessible areas. Challenges discussed included reporting of false AFP cases, late reporting, additional costs required to collect specimens, and the inability to directly supervise the work of the informants.

*Respondent 5: “the major strength really lies on the ability of the informants to be able to navigate into these inaccessible areas, to be able to interact with the caregivers without any problem.”*

### Local level negotiation for evacuation of AFP cases

Most respondents (12/16) discussed the strategy of temporarily evacuating children with suspected AFP for confirmation and investigation. Given that CIIAs were not health workers and often illiterate, and inaccessible areas had no electricity, the most feasible but sometimes dangerous approach for collecting specimens and conducting case investigations and clinical examinations was to bring the patient to an accessible area of Borno. Funds were pre-positioned at LGAs to cover lodging, meals, and medical care costs, which played a large role in persuading families to agree to evacuation. While this strategy greatly improved case investigation, cases were often investigated late after onset due to the challenges of evacuation, including travel by foot or horse-drawn cart. It is also not clear if all children with suspected AFP were evacuated; there was no system in place for recording information about suspected AFP in children who could not be evacuated. Of note, many respondents explained that the work of the CIIAs, including evacuation of cases, required direct negotiation with the insurgents at the local level. Several respondents emphasized the importance of CIIAs having established the trust of local insurgent actors.

*Respondent 12: "The community informants have been able to gain the trust of the community. So, even if a child of a terrorist needs to be evacuated, these guys can still go ahead and do the vaccination, because they have been trusted, they cannot be attacked. But if a soldier, a military man approaches those communities, the terrorists or the bad boys can engage them in a fight."*

### Use of GIS technology

Respondents enthusiastically described the benefits of GIS technology for implementing and monitoring of surveillance in inaccessible areas. The methods of satellite imagery analysis for assessing populations in Borno has been described elsewhere [13]. Before the use of satellite imagery, there was conflicting information on the size and location of populations remaining in inaccessible areas. Satellite imagery allowed estimation of inhabitation, population size and precise location of settlements in the inaccessible areas. Over 12,000 settlements in the inaccessible districts were regularly analyzed using satellite imagery to estimate the inaccessible population, prioritize areas for implementing surveillance and vaccination activities, track progress in reaching the population, and advocate with security forces for support in reaching inaccessible populations if needed.

**Fig. 3:**
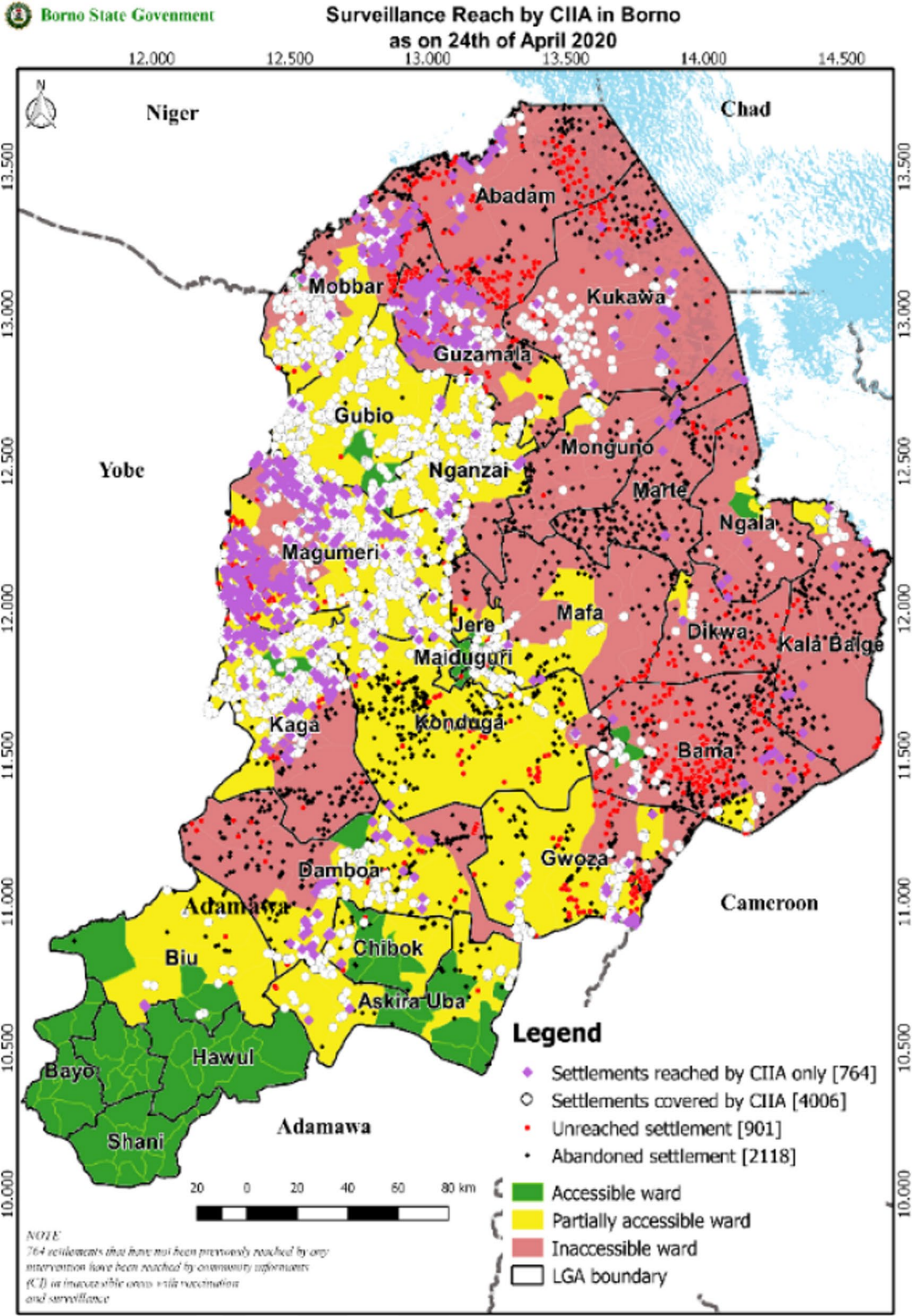
Map of surveillance visits in Borno since 2014 as of April 2020 (provided by the Borno polio Emergency Operations Center)

Most respondents discussed the value of GPS-enabled phones as an accountability tool for tracing and documenting the places CIIAs visited, although several reported logistical difficulties in providing phones to CIIAs. This led to the development of a modified surveillance monitoring system focused on process indicators including the number of settlements reached and the number of AFP cases detected and investigated. The monitoring system relied heavily on GIS technology to regularly map the reach of the program and produce reports for program planning (Fig. 3). A diverse data team worked in an ongoing process of refining the system and analyzing and reporting the monitoring data. The polio Emergency Operations Center in Borno facilitated strong collaboration across organizations involved in the polio program and the humanitarian response.

Respondent 1: *“We use satellite imagery to estimate population, population usually in trapped areas…. And that has really been helpful in the program.”*

Respondent 3: *“The most important tool is the Geo-Location Tracking Systems, which I call the GTS. That shows that the person has been to a settlement. He cannot be somewhere else and then the geo-location system would show somewhere else. So, the next monitoring system is the geo-location monitoring system that is being used to show that they have visited the community itself.”*

*Respondent 16: “so being able to use the tracking phones to add another layer of accountability, I think has been extremely valuable. So you can make sure that if somebody says they reach, they reached a settlement… Well, you can see. Alright. Did you actually go there? Did you actually spend enough time to do what you said you did?”*

## Discussion

### Key findings

Our case study found that the major challenges to standard AFP surveillance activities in conflict-affected areas of Borno state were inaccessibility due to insecurity and the complete destruction of health and communication infrastructure. The most effective strategies to overcome these challenges were the recruitment of community informants with access to inaccessible areas, evacuation of AFP patients for investigation and specimen collection, and use of GIS technology for estimating the population size and location of the inaccessible settlements and tracking surveillance visits in the inaccessible areas. Since 2019 the polio program in Africa has started using GIS more widely for polio surveillance. However, its use is still limited in areas of armed conflict. Implementation of these strategies involves risk and requires a careful balancing of the safety of the local actors with the achievement of public health goals. Although the surveillance data for Borno, as a critical geography, was sufficient for certification of the eradication of indigenous WPV from the World Health Organization (WHO) Region of Africa in August 2020, the remaining challenges include pockets of settlements still unreached by vaccination and surveillance activities, uncertain regularity and quality of surveillance in the inaccessible areas, and challenges with investigating contacts of AFP cases and conducting 60-day follow up examinations when case specimens cannot be promptly collected.

Traditional performance monitoring for polio surveillance relies heavily on tracking the rate of non-polio (NP) AFP detection in children under 15 years of age [35]. However, monitoring this rate is less useful in areas of armed conflict and insecurity because the populations in those areas are often small, with low likelihood of reporting a background NP AFP case every year, of uncertain size and with severely limited health care access. In addition, the NP AFP rate assumes a relatively homogenous level of AFP detection in a given area, and low case detection in inaccessible areas may be masked by high detection in accessible areas within the same administrative area. The risk of assumed homogeneity in the surveillance performance indicators for LGAs within Borno state can be seen in the premature decision by WHO to remove Nigeria from the list of WPV-endemic countries in 2015, after one year without any WPV detection [36]. Revisions in the performance monitoring system for surveillance in inaccessible areas were necessary, focusing on accurately identifying the populations at risk and using process indicators and GPS tracking of surveillance visits in the inaccessible areas.

### Recommendations

To further improve surveillance performance in the inaccessible areas of Borno State, we recommend developing systems to: (1) report and track suspected AFP cases that are not evacuated for investigation; (2) track the regularity of surveillance visits by CIIAs and categorize settlements by frequency of visits; (3) track the collection of specimens from contacts of AFP cases when specimen collection from patients is not timely; (4) continually enumerate the number of children < 15 years of age unreached by surveillance and < 5 years of age unreached by vaccination using GIS tracking data and satellite imagery analysis; and (5) use this AFP surveillance approach to detect other priority diseases in the inaccessible areas.

This study suggests useful approaches for other areas of armed conflict. The progress in Borno required sustained efforts with full financial backing, constant innovation, collaboration among partners, attention to data accuracy, and a focus on accountability and transparency. The use of local-level negotiation by community actors to expand access may be useful in other settings where higher-level negotiations are not successful. Collaboration with security forces can be useful for some areas where civilian staff cannot work safely. Furthermore, the development of novel strategies and monitoring systems demonstrated how a bottom-up approach to partner collaboration in Borno was employed to achieve a common goal through innovation, collaboration, attention to data, and accountability. This approach may serve as a model for how staff from government, international agencies, non-governmental organizations and community members can work together. Finally, GIS is a very powerful tool for assessing inhabitation status of settlements in conflict areas and for tracking interventions.

### Limitations

This study is subject to several limitations. Only publicly available documents were analyzed. The use of a sole researcher to collect data may have led to subjectivity in the sample selection and analysis. As mentioned above, to address this, several strategies to strengthen validity were employed including development of a robust sampling strategy, triangulation among data sources, use of a second coder, member checking, and peer debriefing. The interviews were limited to 16 respondents, although interviews continued until the point of response saturation. In addition, some cadres of staff sought in the sampling frame did not participate. Finally, it would have been useful to directly interview community-level respondents, however because of the vulnerability of that population they were excluded.

## Conclusions

This study found that, even in the most insecure and inaccessible areas of Borno State Nigeria, it was possible to conduct sensitive public health surveillance using modified approaches. In August 2020 the countries of the World Health Organization Africa Region were certified as free from WPV, an achievement that rested largely on the vaccination and surveillance activities conducted in the conflict-affected areas of Nigeria, particularly Borno state [10]. This study revealed a very effective system of collaboration to address an adaptive problem with no easy solutions. The approach used in Borno along with some of the specific strategies of local negotiated access, collaboration with security forces, and use of GIS technology, may be useful for other public health interventions in areas of armed conflict.

## Data Availability

The de-identified datasets used and/or analyzed during the current study are not publicly available due to the sensitive nature of this topic but are available from the corresponding author upon reasonable request. The full report from this study as well as the study protocol are also available from the corresponding author upon reasonable request.

## Footnotes

1 “The sequence of the complete VP1 surface protein coding region is determined by using automated cycle-sequencing procedures described previously [6] and by comparing the resulting sequences with those in a database of all recent poliovirus isolates. The origins and routes of virus importation are then derived from phylogenetic analysis.”

2 Cellular phone networks.
